# Global molecular diversity of Hepatitis E virus in wild boar and domestic pig

**DOI:** 10.1016/j.onehlt.2021.100304

**Published:** 2021-08-09

**Authors:** Maria Casares-Jimenez, Pedro Lopez-Lopez, Javier Caballero-Gomez, Mario Frias, Belen Perez-Hernando, Adeolu Sunday Oluremi, Maria A. Risalde, Inmaculada Ruiz-Caceres, Oluyinka Oladele Opaleye, Ignacio Garcia-Bocanegra, Antonio Rivero-Juarez, Antonio Rivero

**Affiliations:** a Unidad de Enfermedades Infecciosas, Grupo de Virología Clínica y Zoonosis, Instituto Maimónides de Investigación Biomédica de Córdoba (IMIBIC), Hospital Reina Sofía, Universidad de Córdoba (UCO), Córdoba, Spain; b Grupo de Investigación en Sanidad Animal y Zoonosis (GISAZ), Departamento de Sanidad Animal, Facultad de Veterinaria, Universidad de Córdoba (UCO), Córdoba, Spain; c Department of Medical Microbiology and Parasitology, Ladoke Akintola University of Technology, Ogbomoso, Nigeria; d Grupo de Investigación en Sanidad Animal y Zoonosis (GISAZ), Departamento de Anatomía y Anatomía Patológica Comparadas y Toxicología, Facultad de Veterinaria, Universidad de Córdoba (UCO), Córdoba, Spain

**Keywords:** Hepatitis E virus, Wild boar, Pig, Genotype, Prevalence, One health

## Abstract

Our study aim was to describe and characterize the global Hepatitis E virus (HEV) molecular and genotype geographical distribution in domestic pig and wild boar, which could facilitate the traceability of human cases. We performed a systematic sequence search for HEVs identified in domestic pig and wild boar from the available data in GenBank. Only sequences with lengths greater than 300 nt were included. For all sequences, the sequence length, host (i.e., domestic pig or wild boar), country of origin, and HEV genotype/subtype were recorded. Genotypes were assigned by the HEVnet typing tool. The genotype distributions were described by country and host. In countries with sequences available for both species, the genotype coincidences between both animal populations were analyzed. A total of 1404 viral sequences were included: 32.6% from wild boar and 67.4% from domestic pig. Most sequences were consistent with HEV genotype 3 (*n* = 1165). Genotype 4 was represented by 193 sequences, while genotypes 5 and 6 were represented by only 6 sequences. Sequences were identified in 39 countries, which included all continents except Antarctica. The genotypes with a wide distribution were 3a and 3f. Twenty-five countries had sequences that were found only in domestic pig, three countries only in wild boar, and 11 countries had sequences in both populations. In all countries with available sequences in both populations, the same viral genotype was identified. Our study shows that the number of swine HEV sequences is small, which limits direct comparisons with the sequences identified in humans. The global distribution of genotype 3, together with the wide distribution of genotype 4 in Asia, strongly limits the interpretation of the molecular analysis in the absence of an epidemiological survey of the cases. Increased HEV sequencing in swine should be a priority.

## Highlights

• Our study shows that the number of swine HEV sequences is small.
• The global distribution of genotype 3 strongly limits the interpretation of the molecular analysis.
• Increased HEV sequencing in swine should be a priority.

## 1 Introduction

The hepatitis E virus (HEV) represents a major, leading cause of acute hepatitis around the world [1]. The virus belongs to the genus *Orthohepevirus* A and is molecularly organized in a single positive RNA strand of approximately 7.2 kb containing three open reading frames (ORFs) [2]. Based on the viral sequences, the virus can be classified into eight major genotypes, 1 to 8, and 49 subtypes [3]. From a transmission perspective, these genotypes can be divided into two groups: those that exclusively affect humans (e.g., genotypes 1 and 2) and those that affect a wide range of mammalian hosts, including humans (e.g., genotypes 3 to 8) [4]. Genotypes 1 and 2 are mainly present in Asia and Africa (genotype 2 is also reported in Mexico) and produce medium to large outbreaks (epidemic level) that are associated with the consumption of contaminated water [5]. Genotypes 3 to 8 show a worldwide distribution and produce a continuous number of cases (endemic character) [6]. Despite the wide range of mammals that can carry HEVs [7], swine (domestic and feral) are considered to be the principal host and viral reservoir. Consequently, close contact and the consumption of pork and boar meat constitute the main transmission routes of zoonotic HEVs [8].

Information about the affiliation and traceability of human cases is rare because in the majority of cases, the infection source cannot be determined (only suggested) and, consequently, these cases may not be analyzed. For this reason, there are only a few studies that have linked the zoonotic origins of infections from pigs and boars [9; 10; 11; 12; 13]. For this reason, characterizing most viral strains that circulate among animals should be a priority to facilitate the traceability of human cases in the frequent situations in which the exact infection source is unknown. This is a One Health approach for the epidemiological study of HEV infections. Therefore, the aim of our study was to describe and characterize the global HEV molecular and genotype geographical distributions in domestic pig and wild boar, which could facilitate the traceability of human cases.

## 2 Material and methods

### 2.1 Search strategy and criteria

A systematic sequence search of the GenBank database was performed using the terms “Hepatitis E virus pig”, “Hepatitis E virus boar”, “Hepatitis E virus swine”, “Hepatitis E virus wild boar”, “Hepatitis E virus *S. scrofa*”, and “Hepatitis E virus *S. scrofa domesticus*”. Only those sequences with lengths greater than 300 nt were included in this study. This length was selected by following the European Food Safety Agency (EFSA) recommendations [8]. Three independent researchers conducted the search and determined the sequences that matched the study criteria. Duplicate sequences were identified based on the GenBank accession numbers. The database search was conducted during December 2020.

We constructed a database that included information regarding the viral sequences. These data included the sequence identification number (GenBank accession number), sequence length, host (e.g., domestic pig or wild boar), country of origin, HEV genotype and subtype (Supplementary Material).

### 2.2 Genotype assignation and analysis

The sequences were downloaded in FASTA format from GenBank. All FASTA file sequences were used in the HEVnet typing tool (https://www.rivm.nl/mpf/typingtool/hev/), where the genotype and subtype were assigned. This tool shows high consistency with genotype assignments by considering the viral sequences that are proposed as standards (Table 1) [Smith et al., 2020]. Several subtypes were not assigned by standard classification but were proposed by the HEVnet typing tool. These subtypes are noted as (p).

**Table 1:** Proposed standard viral sequences and genotype assignments using the HEVnet typing tool.

Accession number	Genotype assignment by Smith et al [3]	Genotype assignment by HEV Typing tool
M73218	1a	1a
L08816	1b	1b
X98292	1c	1c
AY230202	1d	1d
AY204877	1e	1e
JF443721	1f	1f
LC225387	1g	1g (p)
FJ457024	**1na**	**1h (p)**
MH918640	**1na**	**1e**
KX578717	2a	2a
MH809516	2b	2b
AF082843	3a	3a
AP003430	3b	3b
FJ705359	3c	3c
AF296165–7	**3d**	**3na**
AB248521	3e	3e
AB369687	3f	3f
AF455784	3g	3g
JQ013794	3h	3h
FJ998008	3i	3i
AY115488	3j	3j
AB369689	**3k**	**3n (p)**
JQ953664	**3l**	**3o (p)**
KU513561	**3m**	**3r (p)**
AB290313	**3na**	**3m (p)**
MF959765	**3na**	**3u (p)**
LC260517	**3na**	**3v (p)**
MK390971	**3na**	**3w (p)**
MF959764	**3na**	**3t (p)**
KP294371	**3na**	**3q (p)**
FJ906895	3ra	3ra
AB197673	4a	4a
DQ279091	4b	4b
AB074915	4c	4c
AJ272108	4d	4d
AY723745	4e	4e
AB220974	4f	4f
AB108537	4g	4g
GU119961	4h	4h
AB369690	4i	4i
AB369688	**4na**	**4k (p)**
MK410048	**4na**	**4k (p)**
AB573435	5a	5
AB602441	6a	6
AB856243	6	6
KJ496143	7a	7
KJ496144	7	7
KX387865	**8a**	**7**
MH410174	8	8

Legend: Genotype and subtype discordances are highlighted in bold. Not assigned genotype (na); proposed (p).

The total number of sequences included were reported. The genotype distributions were described by country and host (e.g., wild boar and domestic domestic pig). In countries with sequences available for both species, the coincidences of the genotypes between both animal populations were analyzed.

## 3 Results

### 3.1 Viral sequences

A total of 1404 viral sequences that matched the inclusion criteria were included. Among them, 456 (32.6%) were isolated from wild boar and 948 (67.4%) were isolated from domestic pig. The information for these sequences is described in the Supplementary Material. Only 43 sequences (3.1%) had lengths longer than 7000 bp (considering the entire viral genome), 23 were described in wild boars and 20 in domestic pig. Thirty-three sequences (2.3%) had lengths between 7000 bp and 1000 bp, and 13 were identified in domestic pig and 20 in wild boar. The majority of sequences (*n* = 1328; 94.6%) had lengths between 1000 bp and 300 bp, with 915 belonging to domestic pig and 413 belonging to wild boar.

### 3.2 Viral genotypes

Most sequences were consistent with HEV genotype 3 (*n* = 1165). Genotype 4 was represented by 193 sequences, while genotypes 5 and 6 were represented by only 6 sequences each one. Table 2 shows the genotype and subtype distributions by host. Genotypes 3a (*n* = 232) and 4i (*n* = 74) were the most prevalent among genotypes 3 and 4, respectively. For a total of 40 sequences, the *Orthohepevirus* A genotype could not be assigned.

**Table 2:** HEV genotype distribution in wild boar and domestic pig.

Genotype	Subtype	Global population	Wild boar	Domestic pig
**3**	*3a*	232	46	186
	*3b*	49	37	12
	*3c*	27	24	3
	*3e*	48	18	30
	*3f*	109	32	77
	*3g*	1	0	1
	*3h*	2	0	2
	*3i*	3	3	0
	*3j*	0	0	0
	*3k (p)*	1	0	1
	*3l (p)*	63	27	36
	*3m (p)*	1	0	1
	*3n (p)*	8	0	8
	*3o (p)*	7	0	7
	*3q (p)*	3	3	0
	*3r (p)*	10	9	1
	*3s (p)*	1	1	0
	*3t (p)*	44	44	0
	*3u (p)*	5	5	0
	*3w (p)*	33	31	2
	*3 na*	519	75	444
	***Total***	**1166**	**355**	**811**
**4**	*4a*	12	11	1
	*4b*	26	1	25
	*4c*	12	1	11
	*4d*	38	5	33
	*4e*	2	0	2
	*4g*	9	9	0
	*4h*	4	0	4
	*4i*	74	45	29
	*4 na*	15	1	14
	***Total***	**192**	**73**	**119**
**5**	*5a*	2	2	0
**6**	*6a*	1	1	0
	*6*	3	3	0
**Not assigned**		40	22	18
**Total**		**1404**	**456**	**948**

Legend: Not assigned genotype (na); proposed (p).

Regarding the 43 sequences that covered the entire genome, 36 were consistent with genotype 3 (3b = 5 sequences; 3e = 11 sequences; 3l (p) = 4 sequences; 3a = 2 sequences; 3q (p) = 1 sequence; 3k (p) = 1 sequence; 3g = 1 sequence; 3m (p) = 1 sequence; 3c = 1 sequence; 3u (p) = 1 sequence; 3f = 2 sequence; 3w (p) = 2 sequence; 3i = 1 sequence; 3t (p) = 1 sequence; 3p (p) = 1 sequence; 3o (p) = 1 sequence; one sequence with non-assigned genotype), four with genotype 4 (4b = 2 sequences; one sequence was consistent with genotype 4a and other with genotype 4g), one sequence was consistent with genotype 5, and 2 sequences with genotype 6.

### 3.3 Country distribution

**Fig. 1:**
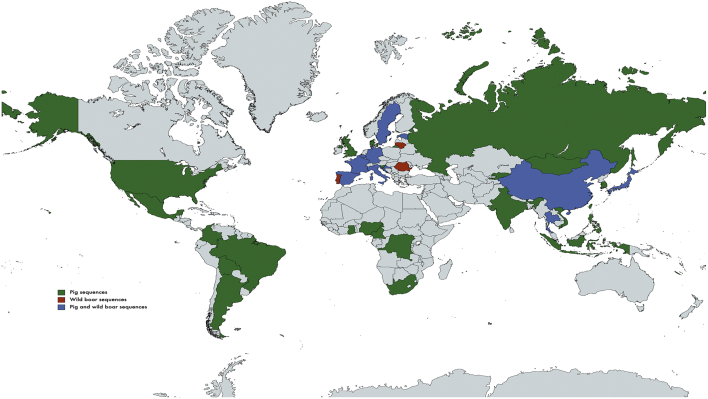
Global distribution of sequences available in domestic pig and wild boar.

Sequences from 39 countries were identified, including all continents except Antarctica (Fig. 1). The majority were from European (*n* = 15) and Asian countries (*n* = 12), while six were from the Americas and Africa, and one was from Oceania (New Caledonia). In Table 3, we summarized the number of sequences by country. Sixteen countries had fewer than 10 sequences available. Twenty-five countries had sequences only in domestic pig, three countries only from wild boar, and 11 countries had available sequences from both animal populations.

**Table 3:** Included HEV sequences by country.

Country	Total sequences included	Wild boar sequences	Domestic pig sequences
Argentina	2	0	1
Belgium	12	8	4
Bolivia	7	0	7
Brazil	59	0	59
Cameroon	2	0	2
China	104	13	91
Colombia	13	0	13
Croatia	28	6	22
Denmark	4	0	4
DR Congo	1	0	1
Estonia	29	7	22
France	68	13	55
Germany	37	37	1
Ghana	8	0	8
India	2	0	2
Indonesia	8	0	8
Italy	173	129	44
Japan	266	155	101
Kyrgyzstan	1	0	1
Lithuania	10	10	0
Mexico	43	0	43
Mongolia	2	0	2
Nigeria	5	0	5
New Caledonia	6	0	6
Philippines	22	0	22
Portugal	1	1	0
Romania	4	4	0
Russia	136	0	136
Sao Tome and Principe	1	0	1
Slovenia	50	0	50
South Africa	7	0	7
South Korea	7	0	7
Spain	52	22	30
Sweden	72	20	52
Taiwan	16	0	16
Thailand	41	1	40
United Kingdom: England	38	0	38
United States of America	45	0	45
Vietnam	21	0	21

### 3.4 Genotype distribution by country

The distribution of HEV genotypes 3 and 4 by country and host is shown in Table 4. Meanwhile, genotype 3 exhibited a wide distribution across continents and countries, and genotype 4 was limited to the Asian countries. Nevertheless, one sequence that was identified in an Italian domestic pig was consistent with genotype 4 (KF939867). The genotypes with wide distributions were 3a and 3f. Among them, genotype 3a was present in 14 countries from Asia, Europe, America and Africa, while genotype 3f was identified in 9 countries from the five continents. The countries with the highest sequence variations were Italy and Japan, with 11 different genotypes each.

**Table 4:**
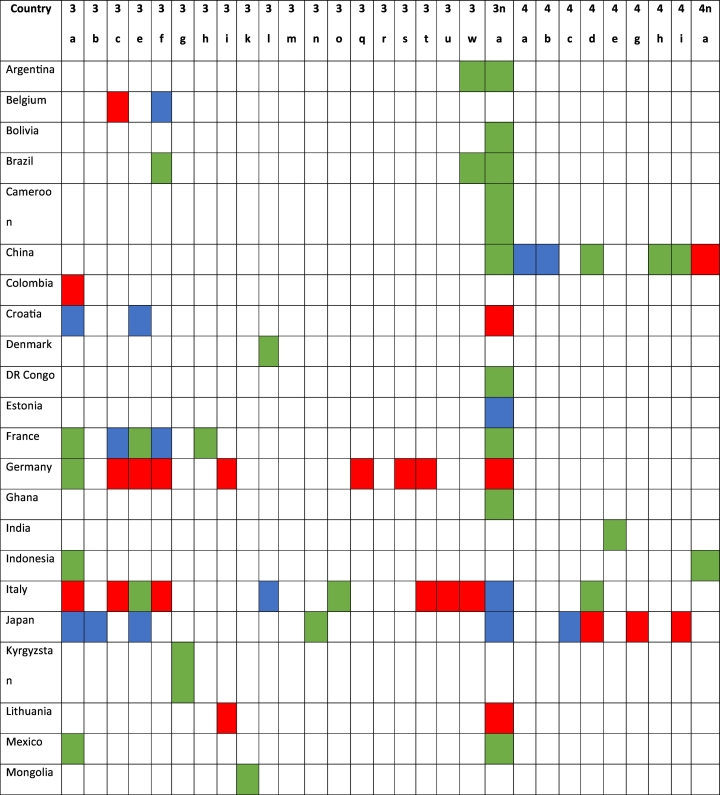

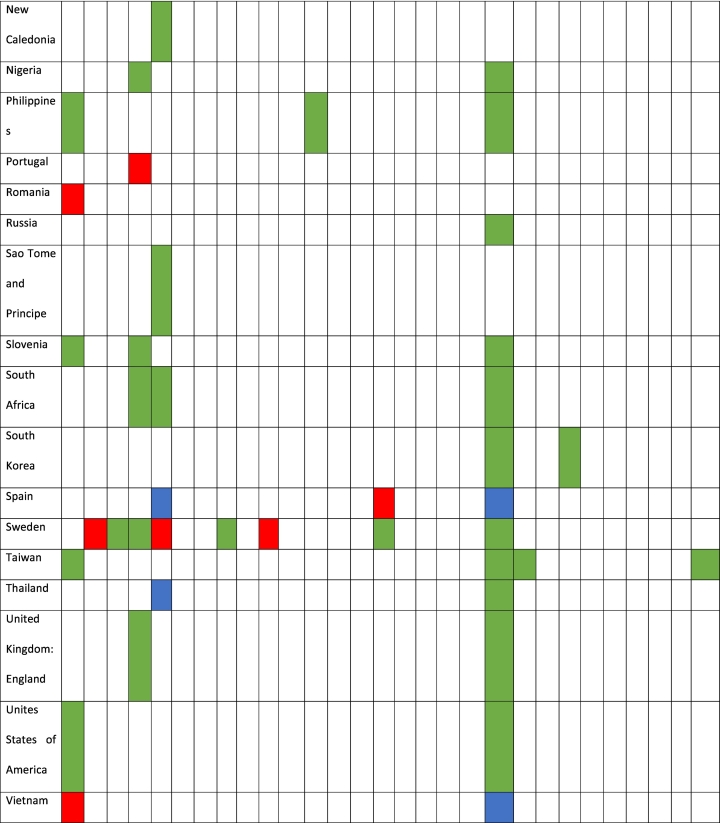
HEV genotype identification by country and host.

Legend: Colored in red (genotype found in wild boar), colored in green (genotype found in domestic pig), and colored in blue (genotype found in wild boar and domestic pig).

Among the 11 countries with available sequences in both domestic pig and wild boar, in 10 countries, the same viral genotypes were identified in both populations (Table 4). These countries were Belgium (genotype 3f); China (genotypes 4a and 4b); Croatia (genotypes 3a and 3f); France (genotypes 3c and 3f); Germany (genotype 3a); Italy (genotype 3l (p)); Japan (genotypes 3a, 3b, 3e and 4c); Spain (genotype 3f); and Thailand (genotype 3f). Estonia had available sequences belonging to genotype 3 in both populations, but in both cases, the subtype could not be assigned.

Genotypes 5 and 6 were detected only in wild boar from Japan. The 40 sequences for which the *Orthohepevirus* A genotype could not be assigned were identified in wild boar and domestic pig from Italy (*n* = 10), wild boar from Japan (*n* = 17), and domestic pig from Russia (*n* = 13) (Supplementary Material).

## 4 Discussion

Zoonotic HEV is considered to be an emerging health issue worldwide. Swine are the main animal host [7] and constitute the main transmission route to humans [14]. The viral sequencing comparison between animal and human strains provides valuable information regarding possible infection routes and, by homology and considering certain risk practices, links the origins of human cases. Nevertheless, direct comparisons are difficult because, although epidemiological investigations of human cases might reveal the consumption of pork or game meat, the viral sequences from these animals or derived food products are usually not available. In this sense, only few works could link the source of the infection by molecular analysis. These studies could compare the strains of patients and hunted wild boar [9,12] or deer [13], assistant to a weeding and roasted piglet consumed during the event [10], or consumers of a specific pork liver sausage [11]. By this reason, in most of the studies the source of the infection cannot be confirmed but suggested. Consequently, no specific measures based on a One Health approach can be taken in a particular country, region, or even at lower levels, such as farms or food chains [15]. Our study provides a global view of molecular HEV in swine, which facilitates the understanding of virus epidemiology. We identified a total of 1403 viral sequences that were isolated from wild boar and domestic pig. This number is extremely small when considering the existing abundant evidence of HEV in this population, because performing the same search in Genbank but only considering those sequences isolated in human, we found a total of 12,606 viral strains. Consequently, only the 10% of the sequences available for HEV have been obtained in wild boar or domestic pig. In this sense, in 2015, an international (mainly European) cross-disciplinary database of HEV sequence data that were retrieved from different sources was established [16]. Of the 1615 sequences available on the HEVnet database, only 75 and 92 have been reported to have animal or food origins, respectively [16]. The lack of a significant number of HEV swine sequences strongly limits the direct comparisons that can be made with the sequences identified in human cases, which has a negative impact on HEV traceability. The sequencing of human cases has obvious epidemiological value to identify potential clusters or the emergence of new genotypes or variants. Nevertheless, in the study of zoonotic viruses, it is mandatory that a similar number of animal origin strains should also be included together with the descriptions of viral sequences that affect humans. Therefore, additional efforts to significantly increase the number of HEV sequences of animal origin are encouraged.

Genotype 3 exhibited a wide distribution and was identified in swine from all continents. For this reason, it was impossible to trace the geographical origin of human cases by considering only the genotypes. In the same way, several subtypes, such as genotypes 3a, 3e and 3f, show global circulation, which also limits the ability to interpret the origins of the infections. These sequences represent 51.9% of the typed genotype 3 strains in our study. These results are consistent with the proportion of human cases that these genotypes represent in several European regions, such as France, Belgium and Italy, which constitute up to 58.2% of the infections [17,18,19]. Consequently, the identification of these genotypes could not exclude the acquisition of the associated infections from outside the country of origin [6]. Furthermore, by considering only the information from these genotypes, it is also difficult to know the infection sources because these genotypes have been isolated from both domestic pig and wild boar in several countries. In this sense, genotype 3a has been identified in both swine populations in Croatia, Germany and Japan, while genotype 3f has been isolated in Belgium, Croatia, France, Spain and Thailand. For this reason, linking the origin and source of an infection by these genotypes requires integrating the deep molecular knowledge of local animal viral strains combined with an exhaustive epidemiological investigation of humans. This was the case for human cases caused by genotype 3f in Japan (genotype not previously identified in this country), which were linked to European pork importation after a sizeable molecular epidemiology investigation [20].

In contrast, there are several genotype 3 subtypes that seem to have specific geographical locations, which could be useful for identifying the infection origins considering their emergence in other locations. In this sense, genotype 3c is located in domestic pig and wild boars from Central Europe. Therefore, the identification of this genotype outside of this region could suggest the importation of this viral strain by live animals or in food products. Scotland is an example of this, where this genotype has emerged in recent years [21]. Because all sequences that have been identified in domestic pig from the United Kingdom are consistent with genotype 3e, the emergence of this genotype in this country probably suggests an association with pork importation from continental Europe. This is information of real value for locating the infection source and controlling the emergence of new viral genotypes. In this way, the emergence of genotype 3e in wild boar and domestic pig from Japan has been linked to the importation of domestic pig raised in Europe [22]. The same explanation could be used for the identification of emergent genotypes between far-flung countries. This is the case for the Philippines and Japan with genotype 3n (p), Spain and Sweden with genotype 3r (p), and Argentina, Brazil and Italy with genotype 3w (p). Further molecular analyses are needed to support this hypothesis.

In contrast to genotype 3, genotype 4 is limited to Asia, with China being the country with the highest prevalence of this genotype and where the majority of its subtypes have been identified. This is consistent with the HEV epidemiology in humans, where genotype 4 seems to be the major cause of HEV infections of zoonotic origin in Asia [23,24,25]. A striking finding is the identification of genotype 4d in domestic pig raised in Italy. Genotype 4 has been previously identified in humans in Italy [26]; however, the presence of this genotype was considered to be important for travel to endemic areas. Nevertheless, the identification of this genotype in Italian domestic pig farms strongly suggests emergence because of the local circulation of this genotype in Italy at the farm level. Longitudinal domestic pig surveys are needed to elucidate whether this emergence is only related to a specific farm or is due to a larger spread.

Finally, genotypes 5 and 6 exhibit very limited circulation and have only been identified in Japanese wild boar [26,27]. The lack of these genotypes in domestic pig strongly suggests limited sympatric contact between the two species. For this reason, although the zoonotic characteristic of genotype 5 was recently demonstrated [28], the absence of this genotype in the species that was associated with greater potential consumption could be related to the fact that, until today, no human cases have been described. On the other hand, there was not identified any case of genotypes 7 or 8, which are demonstrated a cross-species transmission [29]. Because these genotypes have been isolated only camelids from countries where pig are not frequently farmed, this could limit the transmission to swine from the main host [30].

In the present study, we did not perform phylogenetic comparisons among the sequences included in the study. This is because the viral genome region coverts and lengths strongly diverge between countries, genotypes and studies, which limit interpretations of this type of analysis. This point clearly has a negative impact on investigations of the origins of human cases and viral strain importation among countries. More useful information can be extracted only when whole-genome sequences are compared [31]. Nevertheless, this is a complex process that is not available in the majority of veterinary and food safety laboratories worldwide. This is obvious when considering that only 3% of the sequences included in the study had lengths greater than 7000 bp. For this reason, it is urgent to establish a common and single sequencing procedure that might be applied in the majority of laboratories, which would lead to direct comparisons among sequences.

Several limitations should be noted. Firstly, it is possible that the structure of the search did not capture some sequences and, consequently, have not been included in the study. Secondly, the reason to do not assig 40 sequences to any Orthohepevirus A genotype do not imply the identification of undescribed genotype. This could be more related with the sequencing procedure, including a short length sequence or the amplification of a conservated region of the viral genome.

In conclusion, our study shows that the number of swine HEV sequences is small, which limits direct comparisons with those sequences identified in humans. Although genotyping could be useful for determining the origins of human cases under certain conditions, the global distribution of genotype 3 along with the wide distribution of genotype 4 in Asia, strongly limits the ability to interpret the molecular analyses in the absence of an epidemiological survey of the cases. A wide sequencing strategy in swine and their derived products is needed to establish individualized preventive measures that may minimize transmission to humans.

## Conflict of interests

The authors declare that there are no competing interests. The authors or their institution have at no time received payment or services from a third party for any aspect of the submitted work (data monitoring board, study design, manuscript preparation, statistical analysis, and soon).

## Data availability statement

All data generated or analyzed during the study are included in this published article. The datasets used and/or analyzed during the present research project are available as Supplementary Material.

## Author contributions

ARJ designed the study. MCJ and PLL search the sequences. JCG, MF and BP verified those sequences that matched the study criteria and available data. MCJ, PLL and ARJ performed the genotyping analysis. All authors interpreted the data. ARJ and ARR obtained funding. MCJ and ARJ created a draft of the paper. All authors revised the draft critically for important intellectual content. All authors contributed to the article and approved the submitted version.

## Funding

This work was supported by the Ministerio de Sanidad (RD12/0017/0012) integrated in the Plan Nacional de I + D + I and cofinanced by the ISCIII-Subdirección General de Evaluación and the 10.13039/501100008530Fondo Europeo de Desarrollo Regional (FEDER), Fundación para la Investigación en Salud (FIS) del Instituto Carlos III (PI19/00864). ARJ is the recipient of a Miguel Servet Research Contract by the Ministerio de Ciencia, Promoción y Universidades of Spain (CP18/00111). MF is the recipient of a Sara Borrell contract by the Ministerio de Ciencia, Promoción y Universidades of Spain (CD18/00091). JCG is supported by an FPU grant from the Spanish Ministry of Education, Culture and Sport (FPU17/01319). AR is the beneficiary of Contratos para la intensificación de la actividad investigadora en el Sistema Nacional de Salud by the Ministerio de Ciencia, Promoción y Universidades of Spain (INT20-00028). The funders did not play any role in the design, conclusions, or interpretation of the study.

## Appendix A Supplementary data

Viral sequences included in the study Image 1
